# Poly[(μ_3_-4-amino­benzene­sulfonato-κ^3^ *N*:*O*:*O*)(triphenyl­phosphine-κ*P*)silver(I)]

**DOI:** 10.1107/S1600536810024207

**Published:** 2010-06-26

**Authors:** Omid Sadeghi, Mostafa M. Amini, Seik Weng Ng

**Affiliations:** a Department of Chemistry, General Campus, Shahid Beheshti University, Tehran 1983963113, Iran; b Department of Chemistry, University of Malaya, 50603 Kuala Lumpur, Malaysia

## Abstract

In the title 1:1 silver 4-amino­benzene­sulfonate adduct with triphenyl­phosphine, [Ag(C_6_H_6_NO_3_S)(C_18_H_15_P)]_*n*_, the sulfon­ate –SO_3_ unit bridges, through only one O atom, two phosphine-coordinated Ag atoms, forming a centrosymmetric Ag_2_O_2_ rhombus. The Ag^+^ cation adopts a considerably distorted a tetra­hedral coordination. In the crystal, adjacent binuclear mol­ecules are connected into a layer motif through the amino group of the anion; the layers are perpendicular to the *a* axis.

## Related literature

For the synthesis of the silver reactant used in the synthesis, see: Hanna & Ng (1999 ▶); Ng & Othman (1997 ▶). For the crystal structure of 4-amino­benzene­sulfonic acid, see: Banu & Golzar Hossain (2006 ▶); Low & Glidewell (2002 ▶); Rae & Maslen (1962 ▶). For literature on silver 4-amino­benzene­sulfonate, see: Léon (1945 ▶, 1992 ▶); Pan *et al.* (2003 ▶); Schreuer (1999 ▶). For other metal derivatives, see: Brodersen & Beck (2004 ▶); Li *et al.* (2006 ▶); Liu, Ma & Yang (2007 ▶); Liu, Wu *et al.* (2007 ▶); Ou *et al.* (2008 ▶); Wu *et al.* (2008 ▶); Zheng *et al.* (2002 ▶). For a review on metal sulfonates, see: Cai (2004 ▶).
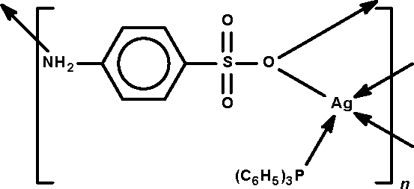


## Experimental

### 

#### Crystal data

[Ag(C_6_H_6_NO_3_S)(C_18_H_15_P)]
*M* *_r_* = 542.32
Monoclinic, 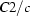

*a* = 28.2593 (15) Å
*b* = 9.4085 (5) Å
*c* = 18.5765 (10) Å
β = 118.229 (1)°
*V* = 4351.6 (4) Å^3^
*Z* = 8
Mo *K*α radiation
μ = 1.12 mm^−1^
*T* = 100 K
0.35 × 0.30 × 0.05 mm

#### Data collection

Bruker SMART APEX diffractometer
Absorption correction: multi-scan (*SADABS*; Sheldrick, 1996 ▶) *T*_min_ = 0.695, *T*_max_ = 0.946
19921 measured reflections
4995 independent reflections
4393 reflections with *I* > 2σ(*I*)
*R*_int_ = 0.038

#### Refinement

*R*[*F* ^2^ > 2σ(*F* ^2^)] = 0.028
*wR*(*F* ^2^) = 0.073
*S* = 1.02
4995 reflections
280 parameters
H-atom parameters constrained
Δρ_max_ = 0.93 e Å^−3^
Δρ_min_ = −0.46 e Å^−3^

### 

Data collection: *APEX2* (Bruker, 2009 ▶); cell refinement: *SAINT* (Bruker, 2009 ▶); data reduction: *SAINT*; program(s) used to solve structure: *SHELXS97* (Sheldrick, 2008 ▶); program(s) used to refine structure: *SHELXL97* (Sheldrick, 2008 ▶); molecular graphics: *X-SEED* (Barbour, 2001 ▶) and *OLEX* (Dolomanov *et al.*, 2003 ▶); software used to prepare material for publication: *publCIF* (Westrip, 2010 ▶).

## Supplementary Material

Crystal structure: contains datablocks global, I. DOI: 10.1107/S1600536810024207/su2190sup1.cif

Structure factors: contains datablocks I. DOI: 10.1107/S1600536810024207/su2190Isup2.hkl

Additional supplementary materials:  crystallographic information; 3D view; checkCIF report

## Figures and Tables

**Table:** 

Ag1—P1	2.3614 (6)
Ag1—O1	2.4252 (15)
Ag1—O1^i^	2.5031 (16)
Ag1—N1^ii^	2.3749 (18)

**Table:** 

P1—Ag1—O1	131.56 (4)
P1—Ag1—O1^i^	124.03 (4)
P1—Ag1—N1^ii^	132.96 (5)
O1—Ag1—O1^i^	80.21 (5)
O1—Ag1—N1^ii^	78.12 (6)
O1^i^—Ag1—N1^ii^	92.43 (6)

Symmetry codes: (i) 
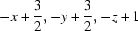
; (ii) 
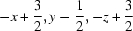
.

**Fig. 1.**
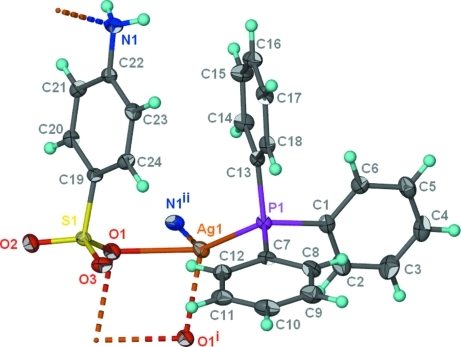
Thermal ellipsoid plot (Barbour, 2001) of a portion of the asymmetric unit of the two-dimensional network structure of the title compound; ellipsoids are drawn at the 70% probability level and H atoms are of arbitrary radius. Symmetry transformation: (i) = 1 - x, 1 - y, 1 - z; (ii) = 3/2 - x, y - 1/2, 3/2 - z.

**Fig. 2.**
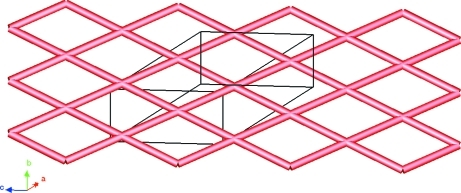
OLEX (Dolomanov et al., 2003) representation of the layer motif in the crystal structure of the title compound.

## supplementary crystallographic information

### Comment

The crystal structure of silver 4-aminobenzenesulfonate, *sulfargenta*, a chemical whose ability to disinfect contaminated water was reported in 1945 (Léon, 1945; 1992), features a polymeric ribbon structure in which the nitrogen and three oxygen atoms are all involved in coordinating to silver centers (Schreuer, 1999; Pan *et al.*, 2003). The 4-aminobenzenesulfonate ion has been studied in other metal salts; the sulfonate part of the ion exhibits diverse coordination modes, as summarized in a review of metal arenesulfonates (Cai, 2004).

Introducing a monodentate ligand such as triphenylphosphine to silver 4-aminobenzenesulfonate should lower the dimensionality (*i.e.*, the adduct should exist as a monomeric molecule) following the suggestion of lowering the dimensionality of the related metal carboxylates by the use of bidentate *N*-heterocycles. However, the hexamethyleneteramine adduct has a layer structure in which only the hexamethylenetetramine ligand participates in *µ*_3_-bridging (Zheng *et al.*, 2002); the 1,1'-(1,4-butanediyl)-bis(imidazole) adduct similarly features an uncoordinated 4-aminobenzesulfonate group (Li *et al.*, 2006). Other bidentate *N*-heterocycles result in silver 4-aminobenzenesulfonate adducts displaying chain or ladder motifs (Liu Ma & Yang, 2007; Liu, Wy *et al.*, 2007; Wu *et al.*, 2008). In the present study, the donor ligand is triphenylphosphine.

In the title 1:1 adduct with triphenylphosphine the sulfonate –SO_3_ group bridges, through only one oxygen atom, two phosphine-coordinated silver atoms to furnish a centrosymmetric Ag_2_O_2_ rhombus (Fig. 1). The silver atom has a tetrahedral geometry as seen from the selected bond distances and angles involving atom Ag1, given in Table 1. In the –SO_3_ portion, one bond is distinctly longer than the other two [1.489 (2) Å compared to 1.444 (2) and 1.457 (2) Å]; the oxygen atom involved in the longer bond is that which bridges the two silver atoms. Sulfanilic acid itself exists as a zwitterion but the longest bond is only slightly longer than the other two [1.476 (1) Å compared to 1.445 (1), and 1.457 (1) Å] (Low & Glidewell, 2002). On the other hand, the bonds are more symmetrical in the two modifications of the monohydrated acid (Banu *et al.*, 2006; Rae & Maslen, 1962).

In the crystal structures of metal 4-aminobenzesulfonates (without other ligands) for which the anion is coordinated to the metal, the amino group is not usually involved in additional coordination. The exceptions are limited to silver (Schreuer, 1999; Pan *et al.*, 2003) and mercury (Brodersen & Beck, 2004) derivatives only. In the crystal structute of the title compound adjacent [Ag(C_6_H_6_NO_3_S)(C_18_H_15_P)]_2_ dimers are connected into a layer motif through the amino moiety. The layers are perpendicular to the *a*-axis of the monoclinic unit cell (Fig. 2), with the aromatic rings of the phosphine ligand protruding into the space between the layers.

### Experimental

Silver acetate (1 mmol, 0.17 g) and triphenylphosphine (2 mmol, 0.53 g) were heated in ethanol (50 ml) until the reactants dissolved completely. Gray insoluble material was removed by filtration and the solvent removed to yield bis(silver acetate^.^2triphenylphosphine) monohydrate sesquiethanol (Hanna & Ng, 1999; Ng & Othman, 1997). The adduct (0.5 mmol, 0.69 g) and 4-aminobenzenesulfonic acid (1 mmol, 0.17 g) were placed in a convection tube; the tube was filled with methanol and kept at 343 K. Colorless crystals were collected after 3 days (m.p. > 573 K).

### Refinement

Hydrogen atoms were placed in calculated positions and treated as riding atoms: C–H 0.95, N–H 0.86 Å with U_iso_(H) = 1.2U_eq_(parent c- or N-atom).

### Crystal data

**Table:**

[Ag(C_6_H_6_NO_3_S)(C_18_H_15_P)]	*F*(000) = 2192
*M**_r_* = 542.32	*D*_x_ = 1.656 Mg m^−^^3^
Monoclinic, *C*2/*c*	Mo *K*α radiation, λ = 0.71073 Å
Hall symbol: -C 2yc	Cell parameters from 8286 reflections
*a* = 28.2593 (15) Å	θ = 2.4–28.2°
*b* = 9.4085 (5) Å	µ = 1.12 mm^−^^1^
*c* = 18.5765 (10) Å	*T* = 100 K
β = 118.229 (1)°	Plate, colorless
*V* = 4351.6 (4) Å^3^	0.35 × 0.30 × 0.05 mm
*Z* = 8	

### Data collection

**Table:**

Bruker SMART APEX diffractometer	4995 independent reflections
Radiation source: fine-focus sealed tube	4393 reflections with *I* > 2σ(*I*)
graphite	*R*_int_ = 0.038
ω scans	θ_max_ = 27.5°, θ_min_ = 1.6°
Absorption correction: multi-scan (*SADABS*; Sheldrick, 1996)	*h* = −36→36
*T*_min_ = 0.695, *T*_max_ = 0.946	*k* = −11→12
19921 measured reflections	*l* = −24→24

### Refinement

**Table:**

Refinement on *F*^2^	Primary atom site location: structure-invariant direct methods
Least-squares matrix: full	Secondary atom site location: difference Fourier map
*R*[*F*^2^ > 2σ(*F*^2^)] = 0.028	Hydrogen site location: inferred from neighbouring sites
*wR*(*F*^2^) = 0.073	H-atom parameters constrained
*S* = 1.02	*w* = 1/[σ^2^(*F*_o_^2^) + (0.0414*P*)^2^ + 3.5194*P*] where *P* = (*F*_o_^2^ + 2*F*_c_^2^)/3
4995 reflections	(Δ/σ)_max_ = 0.001
280 parameters	Δρ_max_ = 0.93 e Å^−^^3^
0 restraints	Δρ_min_ = −0.46 e Å^−^^3^

### Fractional atomic coordinates and isotropic or equivalent isotropic displacement parameters (Å^2^)

**Table:**

	*x*	*y*	*z*	*U*_iso_*/*U*_eq_	
Ag1	0.692581 (6)	0.726456 (18)	0.523729 (10)	0.01618 (6)	
S1	0.80640 (2)	0.94131 (6)	0.63130 (3)	0.01479 (11)	
P1	0.61400 (2)	0.83696 (6)	0.51114 (3)	0.01347 (12)	
O1	0.78580 (6)	0.80116 (17)	0.59157 (9)	0.0175 (3)	
O2	0.86414 (6)	0.94896 (18)	0.66660 (10)	0.0215 (3)	
O3	0.77760 (6)	1.05818 (17)	0.57660 (9)	0.0199 (3)	
N1	0.76252 (7)	1.0121 (2)	0.91345 (11)	0.0172 (4)	
H1A	0.7749	0.9431	0.9478	0.021*	
H1B	0.7287	1.0201	0.8978	0.021*	
C1	0.55223 (9)	0.7331 (2)	0.45987 (14)	0.0152 (4)	
C2	0.54236 (9)	0.6587 (3)	0.38905 (15)	0.0241 (5)	
H2	0.5675	0.6618	0.3687	0.029*	
C3	0.49531 (10)	0.5800 (3)	0.34867 (16)	0.0283 (6)	
H3	0.4881	0.5313	0.2998	0.034*	
C4	0.45886 (9)	0.5712 (2)	0.37823 (15)	0.0236 (5)	
H4	0.4272	0.5157	0.3504	0.028*	
C5	0.46888 (9)	0.6441 (2)	0.44884 (15)	0.0211 (5)	
H5	0.4440	0.6386	0.4697	0.025*	
C6	0.51523 (9)	0.7254 (2)	0.48923 (14)	0.0171 (4)	
H6	0.5217	0.7761	0.5373	0.021*	
C7	0.59882 (8)	1.0046 (2)	0.45534 (12)	0.0149 (4)	
C8	0.54666 (9)	1.0490 (2)	0.40320 (13)	0.0189 (4)	
H8	0.5172	0.9886	0.3932	0.023*	
C9	0.53739 (10)	1.1817 (3)	0.36545 (15)	0.0235 (5)	
H9	0.5017	1.2115	0.3298	0.028*	
C10	0.58010 (10)	1.2702 (2)	0.37993 (15)	0.0228 (5)	
H10	0.5737	1.3612	0.3548	0.027*	
C11	0.63252 (10)	1.2255 (2)	0.43143 (14)	0.0216 (5)	
H11	0.6619	1.2859	0.4410	0.026*	
C12	0.64191 (9)	1.0932 (2)	0.46869 (13)	0.0186 (4)	
H12	0.6777	1.0627	0.5033	0.022*	
C13	0.61624 (8)	0.8853 (2)	0.60765 (12)	0.0141 (4)	
C14	0.63746 (9)	0.7881 (2)	0.67208 (14)	0.0184 (5)	
H14	0.6528	0.7019	0.6663	0.022*	
C15	0.63632 (9)	0.8164 (3)	0.74441 (14)	0.0214 (5)	
H15	0.6501	0.7488	0.7875	0.026*	
C16	0.61496 (9)	0.9438 (2)	0.75397 (14)	0.0197 (5)	
H16	0.6141	0.9630	0.8036	0.024*	
C17	0.59487 (8)	1.0430 (2)	0.69125 (13)	0.0171 (4)	
H17	0.5807	1.1305	0.6981	0.021*	
C18	0.59550 (8)	1.0140 (2)	0.61829 (13)	0.0157 (4)	
H18	0.5818	1.0821	0.5754	0.019*	
C19	0.79126 (8)	0.9533 (2)	0.71350 (12)	0.0132 (4)	
C20	0.82240 (8)	0.8805 (2)	0.78608 (13)	0.0154 (4)	
H20	0.8507	0.8203	0.7906	0.019*	
C21	0.81224 (8)	0.8956 (2)	0.85197 (13)	0.0157 (4)	
H21	0.8335	0.8457	0.9014	0.019*	
C22	0.77081 (8)	0.9840 (2)	0.84538 (12)	0.0144 (4)	
C23	0.73961 (8)	1.0556 (2)	0.77244 (13)	0.0166 (4)	
H23	0.7112	1.1154	0.7677	0.020*	
C24	0.74968 (8)	1.0406 (2)	0.70679 (13)	0.0157 (4)	
H24	0.7282	1.0899	0.6572	0.019*	

### Atomic displacement parameters (Å^2^)

**Table:**

	*U*^11^	*U*^22^	*U*^33^	*U*^12^	*U*^13^	*U*^23^
Ag1	0.01579 (9)	0.01658 (10)	0.01879 (10)	0.00209 (6)	0.01033 (7)	0.00064 (6)
S1	0.0160 (2)	0.0163 (3)	0.0144 (2)	−0.00364 (19)	0.0091 (2)	−0.00339 (19)
P1	0.0141 (2)	0.0126 (3)	0.0154 (3)	0.00022 (19)	0.0084 (2)	0.0003 (2)
O1	0.0188 (7)	0.0178 (8)	0.0184 (8)	−0.0042 (6)	0.0110 (6)	−0.0062 (6)
O2	0.0177 (7)	0.0275 (9)	0.0218 (8)	−0.0059 (6)	0.0114 (6)	−0.0070 (7)
O3	0.0261 (8)	0.0192 (8)	0.0166 (7)	−0.0013 (6)	0.0120 (7)	0.0006 (6)
N1	0.0208 (9)	0.0170 (9)	0.0163 (9)	−0.0019 (7)	0.0109 (7)	−0.0007 (7)
C1	0.0157 (10)	0.0099 (10)	0.0192 (11)	0.0017 (7)	0.0077 (8)	0.0023 (8)
C2	0.0212 (11)	0.0267 (13)	0.0279 (12)	−0.0021 (9)	0.0143 (10)	−0.0087 (10)
C3	0.0255 (12)	0.0268 (14)	0.0290 (13)	−0.0015 (10)	0.0100 (10)	−0.0119 (10)
C4	0.0178 (10)	0.0139 (11)	0.0320 (13)	−0.0007 (8)	0.0060 (9)	0.0003 (9)
C5	0.0182 (10)	0.0173 (12)	0.0293 (12)	0.0006 (8)	0.0124 (9)	0.0052 (9)
C6	0.0178 (10)	0.0153 (11)	0.0179 (11)	0.0014 (8)	0.0082 (9)	0.0018 (8)
C7	0.0204 (10)	0.0133 (10)	0.0145 (9)	−0.0003 (8)	0.0111 (8)	0.0003 (8)
C8	0.0203 (10)	0.0191 (11)	0.0209 (11)	0.0001 (9)	0.0127 (9)	0.0022 (9)
C9	0.0269 (12)	0.0229 (12)	0.0240 (12)	0.0071 (10)	0.0149 (10)	0.0065 (10)
C10	0.0375 (14)	0.0138 (11)	0.0237 (12)	0.0019 (9)	0.0200 (11)	0.0027 (9)
C11	0.0314 (12)	0.0191 (12)	0.0190 (11)	−0.0083 (9)	0.0157 (10)	−0.0046 (9)
C12	0.0212 (10)	0.0196 (11)	0.0166 (10)	−0.0043 (9)	0.0101 (9)	−0.0023 (9)
C13	0.0134 (9)	0.0157 (11)	0.0136 (9)	−0.0005 (8)	0.0067 (8)	−0.0005 (8)
C14	0.0229 (11)	0.0126 (11)	0.0208 (11)	0.0035 (8)	0.0113 (9)	0.0029 (8)
C15	0.0288 (12)	0.0162 (11)	0.0213 (11)	0.0046 (9)	0.0136 (10)	0.0052 (9)
C16	0.0234 (11)	0.0201 (12)	0.0188 (10)	−0.0014 (9)	0.0125 (9)	−0.0015 (9)
C17	0.0182 (10)	0.0129 (10)	0.0229 (11)	−0.0006 (8)	0.0119 (9)	−0.0020 (8)
C18	0.0151 (9)	0.0124 (10)	0.0190 (10)	0.0003 (8)	0.0076 (8)	0.0025 (8)
C19	0.0165 (9)	0.0121 (10)	0.0130 (9)	−0.0030 (8)	0.0086 (8)	−0.0036 (8)
C20	0.0159 (9)	0.0124 (10)	0.0180 (10)	0.0005 (8)	0.0080 (8)	−0.0015 (8)
C21	0.0179 (10)	0.0125 (10)	0.0144 (10)	0.0001 (8)	0.0059 (8)	0.0006 (8)
C22	0.0164 (9)	0.0129 (10)	0.0147 (9)	−0.0043 (8)	0.0080 (8)	−0.0013 (8)
C23	0.0156 (9)	0.0169 (11)	0.0188 (10)	0.0017 (8)	0.0095 (8)	−0.0022 (8)
C24	0.0176 (10)	0.0141 (10)	0.0142 (10)	0.0008 (8)	0.0065 (8)	0.0009 (8)

### Geometric parameters (Å, °)

**Table:**

Ag1—P1	2.3614 (6)	C8—H8	0.9500
Ag1—O1	2.4252 (15)	C9—C10	1.384 (4)
Ag1—O1^i^	2.5031 (16)	C9—H9	0.9500
Ag1—N1^ii^	2.3749 (18)	C10—C11	1.395 (4)
S1—O2	1.4441 (15)	C10—H10	0.9500
S1—O3	1.4565 (17)	C11—C12	1.388 (3)
S1—O1	1.4885 (16)	C11—H11	0.9500
S1—C19	1.774 (2)	C12—H12	0.9500
P1—C13	1.821 (2)	C13—C14	1.396 (3)
P1—C7	1.824 (2)	C13—C18	1.400 (3)
P1—C1	1.826 (2)	C14—C15	1.385 (3)
O1—Ag1^i^	2.5031 (16)	C14—H14	0.9500
N1—C22	1.416 (3)	C15—C16	1.391 (3)
N1—Ag1^iii^	2.3749 (18)	C15—H15	0.9500
N1—H1A	0.8600	C16—C17	1.387 (3)
N1—H1B	0.8600	C16—H16	0.9500
C1—C6	1.391 (3)	C17—C18	1.391 (3)
C1—C2	1.397 (3)	C17—H17	0.9500
C2—C3	1.391 (3)	C18—H18	0.9500
C2—H2	0.9500	C19—C24	1.390 (3)
C3—C4	1.380 (4)	C19—C20	1.393 (3)
C3—H3	0.9500	C20—C21	1.390 (3)
C4—C5	1.385 (3)	C20—H20	0.9500
C4—H4	0.9500	C21—C22	1.394 (3)
C5—C6	1.391 (3)	C21—H21	0.9500
C5—H5	0.9500	C22—C23	1.392 (3)
C6—H6	0.9500	C23—C24	1.384 (3)
C7—C8	1.391 (3)	C23—H23	0.9500
C7—C12	1.398 (3)	C24—H24	0.9500
C8—C9	1.395 (3)		
			
P1—Ag1—O1	131.56 (4)	C9—C8—H8	119.9
P1—Ag1—O1^i^	124.03 (4)	C10—C9—C8	120.1 (2)
P1—Ag1—N1^ii^	132.96 (5)	C10—C9—H9	119.9
O1—Ag1—O1^i^	80.21 (5)	C8—C9—H9	119.9
O1—Ag1—N1^ii^	78.12 (6)	C9—C10—C11	119.9 (2)
O1^i^—Ag1—N1^ii^	92.43 (6)	C9—C10—H10	120.1
O2—S1—O3	114.68 (10)	C11—C10—H10	120.1
O2—S1—O1	111.23 (9)	C12—C11—C10	120.2 (2)
O3—S1—O1	111.38 (9)	C12—C11—H11	119.9
O2—S1—C19	106.66 (9)	C10—C11—H11	119.9
O3—S1—C19	105.65 (10)	C11—C12—C7	120.1 (2)
O1—S1—C19	106.65 (9)	C11—C12—H12	120.0
C13—P1—C7	103.46 (10)	C7—C12—H12	120.0
C13—P1—C1	103.10 (10)	C14—C13—C18	119.0 (2)
C7—P1—C1	104.85 (10)	C14—C13—P1	118.77 (17)
C13—P1—Ag1	114.78 (7)	C18—C13—P1	122.22 (16)
C7—P1—Ag1	113.23 (7)	C15—C14—C13	120.6 (2)
C1—P1—Ag1	115.99 (7)	C15—C14—H14	119.7
S1—O1—Ag1	126.03 (9)	C13—C14—H14	119.7
S1—O1—Ag1^i^	108.65 (8)	C14—C15—C16	120.0 (2)
Ag1—O1—Ag1^i^	99.79 (5)	C14—C15—H15	120.0
C22—N1—Ag1^iii^	108.76 (13)	C16—C15—H15	120.0
C22—N1—H1A	109.9	C17—C16—C15	120.1 (2)
Ag1^iii^—N1—H1A	109.9	C17—C16—H16	119.9
C22—N1—H1B	109.9	C15—C16—H16	119.9
Ag1^iii^—N1—H1B	109.9	C16—C17—C18	119.9 (2)
H1A—N1—H1B	108.3	C16—C17—H17	120.1
C6—C1—C2	119.4 (2)	C18—C17—H17	120.1
C6—C1—P1	122.24 (17)	C17—C18—C13	120.4 (2)
C2—C1—P1	118.36 (18)	C17—C18—H18	119.8
C3—C2—C1	119.3 (2)	C13—C18—H18	119.8
C3—C2—H2	120.4	C24—C19—C20	119.86 (19)
C1—C2—H2	120.4	C24—C19—S1	119.90 (16)
C4—C3—C2	121.3 (2)	C20—C19—S1	120.19 (16)
C4—C3—H3	119.3	C19—C20—C21	120.20 (19)
C2—C3—H3	119.3	C19—C20—H20	119.9
C3—C4—C5	119.3 (2)	C21—C20—H20	119.9
C3—C4—H4	120.3	C20—C21—C22	119.88 (19)
C5—C4—H4	120.3	C20—C21—H21	120.1
C4—C5—C6	120.1 (2)	C22—C21—H21	120.1
C4—C5—H5	119.9	C23—C22—C21	119.58 (19)
C6—C5—H5	119.9	C23—C22—N1	118.87 (19)
C1—C6—C5	120.5 (2)	C21—C22—N1	121.33 (19)
C1—C6—H6	119.7	C24—C23—C22	120.57 (19)
C5—C6—H6	119.7	C24—C23—H23	119.7
C8—C7—C12	119.5 (2)	C22—C23—H23	119.7
C8—C7—P1	122.84 (17)	C23—C24—C19	119.91 (19)
C12—C7—P1	117.63 (16)	C23—C24—H24	120.0
C7—C8—C9	120.3 (2)	C19—C24—H24	120.0
C7—C8—H8	119.9		
			
N1^ii^—Ag1—P1—C13	61.30 (10)	C12—C7—C8—C9	−0.9 (3)
O1—Ag1—P1—C13	−55.43 (10)	P1—C7—C8—C9	176.62 (18)
O1^i^—Ag1—P1—C13	−164.36 (9)	C7—C8—C9—C10	−0.2 (4)
N1^ii^—Ag1—P1—C7	179.81 (10)	C8—C9—C10—C11	0.9 (4)
O1—Ag1—P1—C7	63.08 (9)	C9—C10—C11—C12	−0.5 (4)
O1^i^—Ag1—P1—C7	−45.86 (9)	C10—C11—C12—C7	−0.6 (3)
N1^ii^—Ag1—P1—C1	−58.88 (10)	C8—C7—C12—C11	1.3 (3)
O1—Ag1—P1—C1	−175.61 (9)	P1—C7—C12—C11	−176.36 (17)
O1^i^—Ag1—P1—C1	75.45 (9)	C7—P1—C13—C14	−167.37 (17)
O2—S1—O1—Ag1	179.26 (10)	C1—P1—C13—C14	83.59 (18)
O3—S1—O1—Ag1	−51.46 (13)	Ag1—P1—C13—C14	−43.50 (19)
C19—S1—O1—Ag1	63.33 (13)	C7—P1—C13—C18	15.24 (19)
O2—S1—O1—Ag1^i^	−62.88 (11)	C1—P1—C13—C18	−93.81 (18)
O3—S1—O1—Ag1^i^	66.41 (10)	Ag1—P1—C13—C18	139.11 (15)
C19—S1—O1—Ag1^i^	−178.80 (8)	C18—C13—C14—C15	2.2 (3)
P1—Ag1—O1—S1	−5.52 (14)	P1—C13—C14—C15	−175.26 (18)
N1^ii^—Ag1—O1—S1	−143.61 (12)	C13—C14—C15—C16	−1.4 (4)
O1^i^—Ag1—O1—S1	121.79 (13)	C14—C15—C16—C17	−0.1 (4)
P1—Ag1—O1—Ag1^i^	−127.30 (4)	C15—C16—C17—C18	0.8 (3)
N1^ii^—Ag1—O1—Ag1^i^	94.60 (6)	C16—C17—C18—C13	0.1 (3)
O1^i^—Ag1—O1—Ag1^i^	0.0	C14—C13—C18—C17	−1.6 (3)
C13—P1—C1—C6	10.1 (2)	P1—C13—C18—C17	175.83 (16)
C7—P1—C1—C6	−97.93 (19)	O2—S1—C19—C24	135.67 (17)
Ag1—P1—C1—C6	136.39 (16)	O3—S1—C19—C24	13.22 (19)
C13—P1—C1—C2	−169.57 (18)	O1—S1—C19—C24	−105.38 (18)
C7—P1—C1—C2	82.43 (19)	O2—S1—C19—C20	−41.5 (2)
Ag1—P1—C1—C2	−43.2 (2)	O3—S1—C19—C20	−163.96 (17)
C6—C1—C2—C3	0.9 (4)	O1—S1—C19—C20	77.43 (18)
P1—C1—C2—C3	−179.43 (19)	C24—C19—C20—C21	−0.4 (3)
C1—C2—C3—C4	−1.6 (4)	S1—C19—C20—C21	176.75 (16)
C2—C3—C4—C5	1.1 (4)	C19—C20—C21—C22	−0.1 (3)
C3—C4—C5—C6	0.1 (3)	C20—C21—C22—C23	0.6 (3)
C2—C1—C6—C5	0.2 (3)	C20—C21—C22—N1	−174.11 (19)
P1—C1—C6—C5	−179.41 (17)	Ag1^iii^—N1—C22—C23	−80.1 (2)
C4—C5—C6—C1	−0.8 (3)	Ag1^iii^—N1—C22—C21	94.6 (2)
C13—P1—C7—C8	−91.2 (2)	C21—C22—C23—C24	−0.5 (3)
C1—P1—C7—C8	16.6 (2)	N1—C22—C23—C24	174.28 (19)
Ag1—P1—C7—C8	143.97 (17)	C22—C23—C24—C19	0.0 (3)
C13—P1—C7—C12	86.45 (18)	C20—C19—C24—C23	0.5 (3)
C1—P1—C7—C12	−165.81 (17)	S1—C19—C24—C23	−176.71 (17)
Ag1—P1—C7—C12	−38.43 (19)		

Symmetry codes: (i) −*x*+3/2, −*y*+3/2, −*z*+1; (ii) −*x*+3/2, *y*−1/2, −*z*+3/2; (iii) −*x*+3/2, *y*+1/2, −*z*+3/2.
